# Differential Rickettsial Transcription in Bloodfeeding and Non-Bloodfeeding Arthropod Hosts

**DOI:** 10.1371/journal.pone.0163769

**Published:** 2016-09-23

**Authors:** Victoria I. Verhoeve, Krit Jirakanwisal, Tadanobu Utsuki, Kevin R. Macaluso

**Affiliations:** Department of Pathobiological Sciences, School of Veterinary Medicine, Louisiana State University, Baton Rouge, Louisiana, 70803, United States of America; University of Minnesota, UNITED STATES

## Abstract

Crucial factors influencing the epidemiology of *Rickettsia felis* rickettsiosis include pathogenesis and transmission. Detection of *R*. *felis* DNA in a number of arthropod species has been reported, with characterized isolates, *R*. *felis* strain LSU and strain LSU-Lb, generated from the cat flea, *Ctenocephalides felis*, and the non-hematophagous booklouse, *Liposcelis bostrychophila*, respectively. While it is realized that strain influence on host biology varies, the rickettsial response to these distinct host environments remained undefined. To identify a panel of potential rickettsial transmission determinants in the cat flea, the transcriptional profile for these two strains of *R*. *felis* were compared in their arthropod hosts using RNAseq. Rickettsial genes with increased transcription in the flea as compared to the booklouse were identified. Genes previously associated with bacterial virulence including LPS biosynthesis, Type IV secretion system, ABC transporters, and a toxin-antitoxin system were selected for further study. Transcription of putative virulence-associated genes was determined in a flea infection bioassay for both strains of *R*. *felis*. A host-dependent transcriptional profile during bloodfeeding, specifically, an increased expression of selected transcripts in newly infected cat fleas and flea feces was detected when compared to arthropod cell culture and incubation in vertebrate blood. Together, these studies have identified novel, host-dependent rickettsial factors that likely contribute to successful horizontal transmission by bloodfeeding arthropods.

## Introduction

In the last three decades, several species of *Rickettsia* have emerged as human pathogens, including *Rickettsia felis*, a Gram-negative bacterium predominately described in the cat flea, *Ctenocephalides felis*. Biological characteristics and genomic analysis have led to the classification of *R*. *felis* as a transitional group *Rickettsia*, containing attributes of both insect-borne typhus and tick-borne spotted fever group *Rickettsia* [1]. Human infection correlates with high antibody titers to *Rickettsia* and *R*. *felis* DNA in patients’ sera coincides with clinical features including fever, marked fatigue, headache, generalized maculopapular rash [2]. Since its discovery in cat fleas and the first human case reported from Texas, *R*. *felis* has been recognized as a cosmopolitan pathogen [3–7]. Highlighting its emergent nature in sub-Saharan Africa, *R*. *felis* was detected in a number of flea species [8–10] and recently identified as a common (3–15%) cause of fever among febrile patients [3, 11, 12]. In the Americas, *R*. *felis* rickettsiosis is associated primarily with suburban foci corresponding with the detection of rickettsial DNA in a variety of peri-domestic vertebrate hosts and their fleas [13–16].

Transmission of insect-borne rickettsial pathogens such as louse-borne *Rickettsia prowazekii*, the agent of epidemic typhus, to vertebrate hosts by bloodfeeding arthropods occurs through inoculation of infectious insect feces into the vertebrate host [17]. Similarly, the spread of flea-borne *Rickettsia typhi*, the agent of endemic typhus, involves pathogen transmission to vertebrate hosts via infected flea feces, with subsequent oral transmission during feeding made possible by a sustained extrinsic incubation period in the flea host [18]. The association of *R*. *felis* with cat fleas has facilitated a number of studies examining the transmission mechanisms employed. Indeed, the presence of *R*. *felis* in cat flea salivary glands [19] supports the identification of oral transmission during feeding on vertebrate hosts [20, 21] and the ability of co-feeding adult fleas to transmit the pathogen between infected and uninfected fleas [22, 23]. Although a role for *R*. *felis*-infected flea feces in vertebrate or inter-arthropod transmission is undefined, detection of transcriptionally active rickettsiae by qPCR in cat flea feces post-exposure to an infectious bloodmeal suggests that fecal transmission is also employed by this flea-borne pathogen [24]. Despite the recognition of multiple routes, the molecular determinants of insect-borne transmission of rickettsial pathogens are not known.

In addition to its emergence as a flea-borne rickettsial pathogen, several genotypes of *R*. *felis* have been identified in a number of other arthropod species [25]. In *Liposcelis bostrychophila*, also called psocids or common booklice, the presence of vertically maintained *R*. *felis* infection is associated with reproductive manipulation (parthenogenesis) and a positive fitness effect for the arthropod host [26, 27]. Conversely, in cat fleas, vertical transmission of *R*. *felis* is highly variable; a fitness effect has not been described [24]. Thus, it might be expected that there are genetic determinants in individual rickettsial strains that contribute to arthropod host colonization. However, genome comparison of cat flea (*R*. *felis* str. LSU)- and booklouse (*R*. *felis* str. LSU-Lb)-derived isolates revealed that the strains are closely related. While host-specific differences were evident, no distinct gene or set of genes that could be attributed to pathogenicity or horizontal transmission were identified [28]. In the absence of a clear genetic origin for rickettsial transmission by hematophagous arthropods, there exists the potential for a transcriptional basis which reflects both rates of transcription and decay of transcripts identified by RNAseq and qPCR, which is essential to horizontal transmission. We hypothesized that cues associated with hematophagous arthropod infection would induce a host-dependent *R*. *felis* transcription profile. Using next generation sequencing, specifically RNAseq, differentially transcribed genes were identified and host-specific transcription profiles by *R*. *felis* in hematophagous (cat flea) and non-hematophagous (booklice) arthropod hosts described. The expression of a subset of identified genes was further examined in both *R*. *felis* strains in a flea infection bioassay (culture, incubation with blood, and arthropod hosts). A distinct transcription profile was associated with both *R*. *felis* strains in the flea host, but not in culture or blood. The host-dependent transcription profile of *R*. *felis* provides novel insight into the molecular basis for rickettsial virulence and horizontal transmission.

## Materials and Methods

### Arthropod maintenance, *Rickettsia* propagation, and infection bioassays

Both booklice (*L*. *bostrychophila)* and cat flea (*C*. *felis* colonies) were maintained at LSU-SVM as previously described [22, 29]. Briefly, booklice were obtained from shredded corn cobs (Harlan Laboratories) and reared in shredded corn cobs with fine Caribbean sand and flea larval diet [29]. Commercially available cat fleas were purchased (Elward II) and maintained on an artificial host with bovine blood (Rockland) [30]. *R*. *felis* str. LSU and *R*. *felis* str. LSU-Lb, which were originally isolated from the Louisiana State University cat flea colony [31] and booklice [29], respectively, were cultured in an *Ixodes scapularis*-derived cell line (ISE6) [31]. As previously described (25), *R*. *felis* was cultured in ISE6 cells in a humidified incubator with 5% CO_2_ at 32°C. A modified L15B medium containing 10% tryptose phosphate broth (Sigma) and 10% heat-inactivated fetal bovine serum (HyClone) was used for cell maintenance. Viability of *R*. *felis* was determined using the BacLight viability stain kit (Life Technologies) prior to cat flea infection. For infection of cat fleas with *R*. *felis*, partially purified *R*. *felis* from ISE6 cells were resuspended in bovine bloodmeal (1 ml) at the concentration of 1 x 10^10^ rickettsiae per ml and were used for feeding cat fleas as previously described [20, 22]. Briefly, cat fleas were allowed to feed on infected bloodmeals utilizing an artificial host (19) for 36 hours. After infection of cat fleas with *R*. *felis*, infected bloodmeal, feces and cat fleas were collected. Cat fleas were cleaned by sequential surface washing with 10% bleach for 5 min, 70% ethanol for 5 min repeated 3 times, followed by sterile distilled water. Flea salivary glands and guts were dissected out in sterile PBS and the tissues were stored in TRIzol (500 ml) at -80°C until RNA isolation.

### RNA isolation and RNAseq

Total RNA from flea salivary glands and guts or booklice was isolated with TRIzol (Invitrogen) according to manufacturer’s protocol followed by phenol/chloroform purification. The RNA extracted from cat flea tissues and booklice were quantified by spectrophotometry. Eukaryotic and bacterial rRNA were depleted from the total RNA by using the Ribo-Zero Magnetic Gold Kit and Ribo-Zero Magnetic Kit-Gram Negative Bacteria (Epicentre). The remaining RNA was further treated with a NEBNext Poly(A) mRNA Magnetic Isolation Module (New England BioLabs) to deplete eukaryotic mRNA. The resulting RNA was ethanol precipitated and resuspended in 14 μl of RNase-free water. Removal of rRNA and presence of *R*. *felis* mRNA were validated by qPCR using specific primers for 18s rRNA [24] and salivary antigen [For 5’-TGCAGCAGGCCAATGAAT-3’ and Rev 5’-GGAACTATTTCCGTAGGACGAG-3’ (AF102502)] for cat fleas, 18S rRNA for booklice [18sLbFor 5’-TGGAGCTTGGGGCTTAAT-3’ 18sLbRev and 5’-CACCACCACCCACTAAATCA-3’(FJ196622)] and *R*. *felis* 17 kDa surface antigen [24]). An estimated 90% reduction of host transcripts was determined by qPCR before proceeding. No reverse transcriptase controls (water was added instead of reverse transcriptase) were performed to confirm an absence of genomic DNA (gDNA). The transcriptome library was prepared through fragmentation, hybridization, reverse transcription and amplification of cDNA from 100 ng of purified and enriched RNA using the Ion Total RNAseq Kit v2 (Life Technologies) and qualitatively assessed by Agilent Bioanalyzer. The sequencing was carried out with an Ion Torrent Personal Genome Machine (PGM™) System on a 316 chip.

Three independent infection assays were carried out in fleas and compared to three independent preparations of constitutively infected psocids by RNAseq. All data sets have been deposited into the NCBI Sequence Read Archive under bioproject accession PRJNA285354. Sequencing files from *R*. *felis* str. LSU infecting cat fleas are archived under accessions SRR2049306, SRR2051064, and SRR2051066. Sequencing files from *R*. *felis* str. LSU-Lb infecting booklice are archived under accessions SRR2051067, SRR2051068, and SRR2051069.

#### Bioinformatic analysis

Analysis of the resulting reads was facilitated by the Ion Torrent pipeline v2.2.1. After determining quality metrics and filtering low quality reads, alignment to the reference genome for *R*. *felis* URRWXCal2 was performed with TMAP using default settings. Transcript assembly was performed using Cufflinks v2.0.2 (76) and differential transcript analyses comparing rickettsial transcription in cat fleas versus booklice were performed using CuffDiff v2.0.2 [32]. Tuxedo Suite programs were run using the public server of Galaxy (http://usegalaxy.org) [33–35]. Cufflinks was run with the following parameters: -q–I 300000 –f 0.100000 –j 0.150000 and read counts were reported as fragments per kilobase of transcript per million mapped reads (FPKM) with no normalization based on library size. CuffDiff was run with the following parameters: -q–p 8 –c 10 –FDR .050000. FPKM counts were normalized by CuffDiff using the median of the geometric means of the fragment counts for the libraries and subsequent fold-change comparisons were reported as log2(Fold-change). Genomic coordinates designated as transcriptionally increased in cat fleas as compared to booklice were annotated by BlastN search.

### Quantitative PCR (qPCR)

Selected significantly expressed rickettsial transcripts identified by RNAseq with previous virulence association in other bacteria were confirmed quantitatively by qPCR. Infection experiments for qPCR validation were performed separately from the RNAseq infection experiments. Briefly, total RNA was extracted using TRIzol (Invitrogen) according to manufacturer’s instructions, treated with TurboDNase (Ambion), cleaned using the Zymo Clean and Concentrator-5 kit (Zymo). Total purified RNA (50 ng) was reverse transcribed using the iScript cDNA Synthesis kit (Bio-Rad). cDNA was amplified using specific primers for individual target genes. Sequences of primers (Integrated DNA Technologies) for genes of interest (GOI) are listed in **Table 1**. Each 35 μL qPCR reaction was composed of 5 μL of cDNA template, 17.5 μL of LightCycler 480 SYBR Green I Master (Roche), 1 μL of each 10 mM primer and DNase/RNase-free, distilled water. 10 μL of PCR reaction mix was assessed in triplicate on a 384-well plate and amplified using a Light Cycler 480 Real-Time PCR system (Roche). The cycling parameters were 95°C for 10 min, then 45 cycles at 95°C for 10 sec, 55°C for 30 sec, and 72°C for 1 sec, followed by cooling down to 40°C for 30 sec. No reverse transcriptase controls (water was added instead of reverse transcriptase) were performed to confirm the absence of genomic gDNA. Serial dilutions of the pCR4-Topo plasmid containing a portion of individual target genes were assessed in parallel, serving as a standard for concentration analysis. To determine the quantity of rickettsiae in the samples, DNA was extracted from the same samples after RNA isolation and the number of rickettsial genomic equivalents (copies of 17-kDa antigen gene; *Rf*17kDa) was quantified by qPCR [24]. The relative gene expression was quantified as a ratio of target cDNA sequence to copy number of *Rf*17kDa gene, providing the cDNA copy number per individual rickettsia [36]. Data are given as mean ± standard error of the mean. Statistical analyses for qPCR validation of RNAseq were performed by t-test with a p value of ≤ 0.05 considered significant. Additionally, a one-way ANOVA was performed with the flea infection assays, followed by the Tukey’s post-hoc test with a p value of ≤ 0.05 considered significant. The infection experiments were performed twice independently.

**Table 1:** Primers for qPCR validation of RNAseq genes of interest (GOI). Gene sequences from URRWXCal2 *R*. *felis* reference genome (accession CP000053.1, CP000054.1) were used for primer design.

GOI	Forward Primer (5’-3’)	Reverse Primer (5’-3’)	References
*lpxB*	ACCGTCAGTTTGGGCATATAA	CAATCAAGTCCGAGCCTAGTAAA	This study
RF0211	CGTAATAGCACTCGGAGAACAA	CAAGGGCGGTAGAAACTGAA	This study
RF0580	ACTCGCTTACGGCGATCTTA	GCTGTTTCTCCACGCTCATT	This study
*virB4_2*	TATGGCTAGCTTTGCCTCAC	GCGTACCCGAAGTAGTATCAAG	This study
*trmE*	GCAGAAGGGATAGCCGATTT	GCTGAGTGCGCCAGTTATTA	This study
*uvrA*	TGAATGTCGGGCTTGATTATCT	CCGCTAAGTCCTGAACCTATTT	This study
*relB2*	CAGAAGATATAAAGACAGAAGCAGAA	GCAAACCGCCGCTATTTATAC	This study
*Rf*17kDa	AGGACAGCTTGTCGGAGTAGG	ACGCCATTCTACGCTAGTGC	[21]

## Results

### RNAseq comparison of *R*. *felis* str. LSU and *R*. *felis* str. LSU-Lb

**Fig 1:**
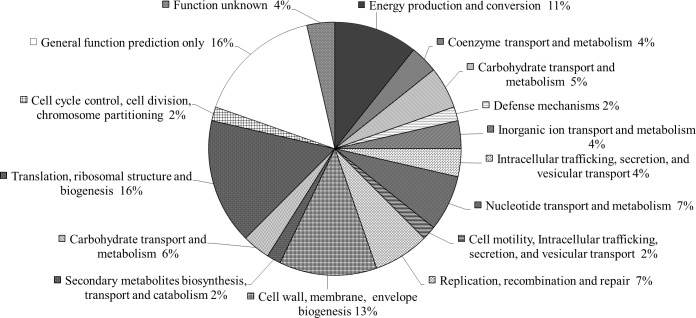
Clusters of Orthologous Groups (COG) containing *R*. *felis* genes upregulated in the cat flea as compared to booklice. COG assignments shown are determined from the URRXWCal2 genome annotation and are represented as the percentage of total differentially upregulated transcripts in fleas.

To investigate *R*. *felis* transcription profiles in hematophagous and non-hematophagous arthropod hosts, three independent infection assays were carried out in fleas and compared to three independent preparations of constitutively infected psocids. The sequencing libraries resulted in 79.6-145bp average read lengths. For each analysis, an average of 30,145 reads (range: 10,671–68,334) were acquired for *R*. *felis* str. LSU in fleas compared to an average of 13,265 reads (range: 8,263–18,287) acquired for *R*. *felis* str. LSU-Lb in psocids. Combined, 115 differentially expressed transcripts in fleas were identified with an average run coverage between 11–27% of the total genome and with an average depth between 0.2–1.8x. Among the 115 differentially expressed genes, 63 genes had higher transcriptional levels in *R*. *felis* within fleas in comparison to the booklice. These differentially expressed genes in fleas were blasted against the *R*. *felis* genome URRWXCal2 and putative assignments were given to 80.9% (51/63) of the genes with the remainder consisting of hypothetical proteins **(S1 Table**). Putatively assigned and hypothetical proteins were further characterized by cluster of orthologous group classification **(Fig 1**).

**Fig 2:**
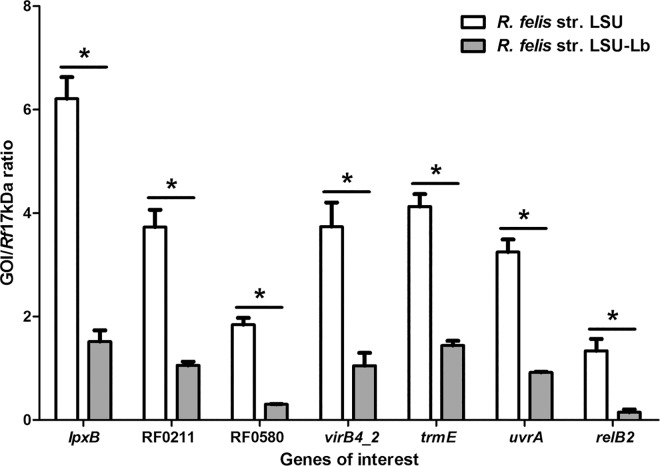
qPCR validation of a subset of upregulated genes determined by RNAseq in *R*. *felis* str. LSU infecting cat fleas as compared to *R*. *felis* str. LSU-Lb constitutively infecting booklice. Copy number for each transcript is divided by the copy number of the *Rf*17kDa gene. Data shown are mean ratio of transcript per rickettsia (GOI/*Rf*17kDa) from two independent experiments. The asterisk denotes a significant difference between the two strains of *R*. *felis* for each target gene as determined by t-test with p ≤0.05 considered significant. Error bars represent the standard error of the mean.

Further analysis identified common transcripts significantly upregulated among three RNAseq replicates, comparing *R*. *felis* str. LSU infecting flea-associated transcripts to *R*. *felis* str. LSU-Lb infecting psocid-associated transcripts. Of the commonly identified upregulated transcripts in fleas, seven genes (*lpxB*, RF0211, RF0580, *virB4_2*, *trmE*, *uvrA*, *and relB2)* were chosen for both validation by qPCR and for further analysis in the flea infection bioassay based on previous virulence association in other bacteria (**Table 2)**. For these selected genes, the differential expression was quantitatively assessed using qPCR to generate the ratio of cDNA copies per rickettsial genomic equivalents. The quantitative analysis confirmed significant increases in rickettsial transcription of the seven commonly identified genes in *R*. *felis* str. LSU-infected fleas compared to *R*. *felis* str. LSU-LB-infected psocids (**Fig 2**). The qPCR transcript analysis confirms that these target *R*. *felis* transcripts are upregulated in hematophagous fleas, compared to non-hematophagous psocids, validating the RNAseq differential transcription analysis.

**Table 2:** Selected *R*. *felis* transcripts with increased expression in cat fleas as compared to booklice chosen for validation by qPCR and transcriptional analysis in the flea infection bioassay. Fold change is reported as the greatest fold-change identified by RNAseq across three biological replicates.

*Rickettsia* ORF	Log_2_(Fold- change)	Gene	Annotation	COG
RF 0519	10.8	*lpxB*	lipid-A-disaccharide synthase	COG0763M
RF 0211	5.8		LolC/E lipoprotein releasing system, transmembrane protein	COG4591M
RF 0580	3.5		ankyrin repeat-containing protein	COG0666R
RF 1250	3.6	*virB4_2*	Type IV secretion/conjugal transfer ATPase	COG3451U
RF 1214	6.5	*trmE*	tRNA modification GTPase	COG0486R
RF 1324	7.3	*uvrA*	excinuclease ABC subunit A	COG0178L
RF 0827	1.7 e+308*	*relB2*	DNA-damage-inducible protein J	COG3077L

*Expression of *relB2* in booklice was below the limit of detection, but the positive fold change was determined statistically significant by CuffDiff.

### Expression of putative bacterial virulence determinants by *R*. *felis* strains during flea infection

**Fig 3:**
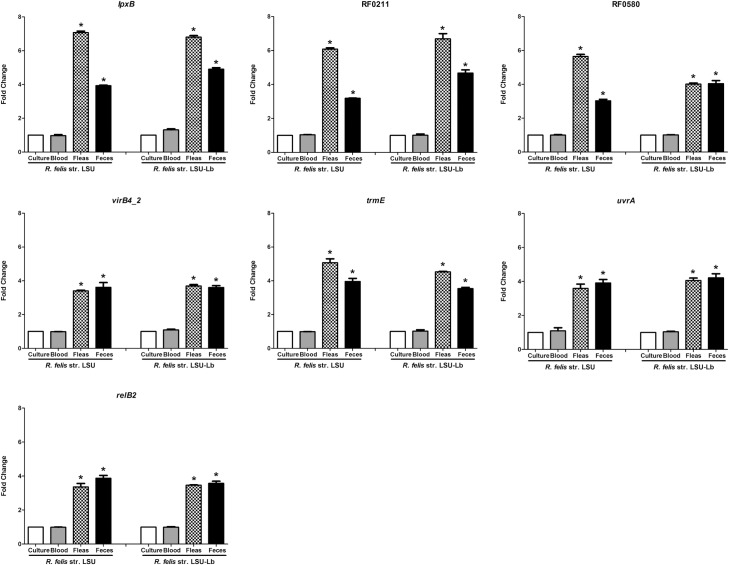
Expression of selected genes of interest by both *R*. *felis* str. LSU and *R*. *felis* str. LSU-Lb during the flea infection bioassay. Cultured *R*. *felis* (in ISE6) was added to bovine blood and fleas were exposed for 36 hours. Cultured bacteria, infectious bloodmeal, fleas, and flea feces were then collected for qPCR. Transcript expression of each gene is divided by the number of rickettsiae in each sample (GOI/*Rf*17kDa) and compared to the ratio of GOI/*Rf*17kDa in ISE6 cells. The fold-change between treatment(s) and culture for the respective *R*. *felis* strains are calculated using two independent experiments. The asterisk denotes significant expression as determined by one way ANOVA followed by a Tukey’s post-hoc test with a p ≤0.05 considered significant. Error bars represent the standard error of the mean.

To determine if transcription of differentially regulated genes (**Table 2**) is specific to the infection of the hematophagous arthropod, transcript levels of *R*. *felis* in adult fleas and also viable *R*. *felis* excreted by infected fleas in feces were compared to *R*. *felis* transcription when rickettsiae were maintained in arthropod cell culture or incubated with blood alone. RNA was isolated from rickettsiae at each stage of the flea infection bioassay (ISE6 cell culture, infected bloodmeal, fleas, and feces). Transcription was then quantified by qPCR, and expression at each stage was compared to transcription in ISE6 culture. As shown in **Fig 3**, transcription of all the genes remain unchanged in the blood as compared to culture. In cat fleas exposed to an infectious bloodmeal of *R*. *felis* str. LSU for 36 hours, *lpxB*, RF0211, RF0580, *virB4_2*, *trmE*, *uvrA* and *relB2* transcription was significantly increased when compared to culture. Likewise, transcription of the seven selected genes was significantly increased in feces from *R*. *felis* str. LSU exposed fleas compared rickettsiae maintained in cell culture.

The two strains of *R*. *felis* used in this study were isolated from very distinct hosts [29, 31]. The previous experiment demonstrated that *R*. *felis* str. LSU isolated from fleas produced a transcription profile dependent on the hematophagous arthropod association. To determine if this was a strain-dependent characteristic, host-dependent transcription of booklouse-derived *R*. *felis* str. LSU-Lb was assessed in the flea infection bioassay, including ISE6 cell culture, infected bloodmeal, fleas, and feces. Similar to *R*. *felis* str. LSU, significant increases in transcription of *lpxB*, RF0211, RF0580, *virB4_2*, *trmE*, *uvrA*, and *relB2* were identified for *R*. *felis* str. LSU-Lb in exposed fleas and resultant feces, as compared to culture in ISE6 cells (**Fig 3**). Considered with the results for *R*. *felis* str. LSU in the flea infection bioassay, the increased transcript expression by *R*. *felis* LSU-Lb in fleas suggest that *R*. *felis* transcription of selected genes occurs in a host-dependent manner and is independent of the origin of the *R*. *felis* strain.

## Discussion

The combination of pathogenicity of bacterial isolates and transmission modalities in the maintenance of pathogens in arthropod vectors are crucial factors which influence the epidemiology of the emerging *R*. *felis* rickettsiosis [8–10]. Variable vertical and horizontal transmission success of the agent by the hematophagous vector, the cat flea, occurs with no appreciable effect on flea fitness [20, 22]. Additionally, *R*. *felis* has been detected within multiple arthropod hosts including other species of fleas, and of particular interest, the non-hematophagous booklouse *L*. *bostrychophila* [26, 29]. Booklouse-associated *R*. *felis* is maintained 100% vertically and acts as a reproductive manipulator inducing parthenogenesis, and removal of the bacteria from the host decreases booklouse fitness [26, 27]. Host-specific vertical maintenance of *R*. *felis*, specifically the variable vertical transmission of *R*. *felis* in cat fleas, suggests the necessity of bacterial reintroduction into cat flea populations [21, 24]. While the presence of *R*. *felis* in multiple arthropods may be facilitated by feeding on a rickettsemic host or indirectly cofeeding with infected arthropods on vertebrate hosts [22, 25], the isolation of genetically similar *R*. *felis*-like organisms (RFLOs) in cofeeding arthropods confound the epidemiology of *R*. *felis* [37]. There is a paucity of transmission studies investigating the complex interaction and transmission of *R*. *felis* and RFLOs in arthropod populations. Most notably missing from rickettsial biology is the identification of transmission determinants differentially encoded in the genomes of *Rickettsia* or expressed differentially in specific hosts to facilitate transmission. Recent genome sequencing and comparison of *R*. *felis* str. LSU and *R*. *felis* str. LSU-Lb assessed the genomic diversity of two *R*. *felis* strains isolated from arthropods with different transmission modes [28]. While genomic divergence was defined between the two strains, no singular genetic determinant or group of determinants (*e*.*g*. plasmids) were identified which would explain known transmission and pathogenic differences. As an extension of the genomic sequencing, we utilized next-generation sequencing methodologies to compare the transcription of *R*. *felis* str. LSU and *R*. *felis* str. LSU-Lb in cat fleas and booklice, respectively, seeking to identify a panel of putative transmission determinants.

The interaction between rickettsial pathogens and their arthropod hosts has been examined previously with the use of microarrays and next-generation sequencing. Microarrays were first used to determine transcriptional differences associated with temperature change and bloodfeeding of *Amblyomma aureolautum*, the yellow dog tick, infected with *Rickettsia rickettsii* [38]. Briefly, five-fold more differentially expressed rickettsial transcripts were observed after bloodfeeding, in contrast to temperature conditions. Notably, the transcriptional profile of bloodfeeding alone and bloodfeeding combined with a temperature shift were similar, suggesting bloodfeeding induces major transcriptional changes by *R*. *rickettsii* [38]. Also, next-generation Illumina sequencing was used previously in conjunction with proteomic analyses to assess the transcriptional response of *Anaplasma phagocytophilum* in the salivary glands of the tick species *I*. *scapularis* after 48 hours of feeding [39]. Using selective depletion methods prior to sequencing, *A*. *phagocytophilum* revealed a pattern of increased transcription and translation machinery coupled with reduced replication and energetics machinery during a time when horizontal transmission most likely occurs. Overall, transcription and translation of proteins targeted to the cell membrane of *Anaplasma* were increased. Together, these two transcriptomic studies demonstrate the complex relationship between Rickettsiales and their bloodfeeding arthropod hosts. The current study builds on these previous studies in order to move towards identifying potential horizontal transmission determinants by comparing the transcription of two similar, but divergent strains of *R*. *felis* in their respective arthropod hosts.

Using an RNAseq analysis of *R*. *felis* str. LSU and str. *R*. *felis* LSU-Lb infected fleas and booklice, respectively, the transcriptional profiles of strains of bacteria infecting their arthropod hosts were compared. The sequencing runs yielded a coverage and depth which is comparable to the Illumina sequencing of *A*. *phagocytophilum* [39]. The *R*. *felis* strain RNAseq differential analysis yielded a total of 115 significantly differentially expressed transcripts. For the purpose of identifying potential *R*. *felis* virulence and transmission determinant associated with the hematophagous arthropod, our subsequent analyses focused on transcripts significantly upregulated in the flea. Specifically there were 63 transcripts significantly upregulated by *R*. *felis* str. LSU in fleas as compared to booklice constitutively infected with *R*. *felis* str. LSU-Lb. These genes were analyzed into COGs with the most represented groups relating to i) cell wall/membrane/envelope biogenesis, ii) energy production and conversion, iii) translation, ribosomal structure and biogenesis, iv) replication, recombination and repair, and those with v) general predicted function or hypothetical proteins. From this panel, selected genes of interest include *R*. *felis* genes differentially upregulated in fleas as compared to booklice that have a previous virulence association in other bacteria. These factors expressed by *R*. *felis* in the cat flea are potentially required for virulence and horizontal transmission. Putative virulence associated genes which were upregulated included lipopolysaccharide (LSP) biosynthesis genes, a Type IV secretion system component and putative effector, ABC transporter systems, and toxin-antitoxin system components. The expression of hypothetical proteins is a common factor in all expression experiments to date. Collectively, 27% of the genes identified in this study have either a generalized function or no function (hypothetical protein) assigned in the reference URRWXCal2 genome (accessions: CP000053.1 and CP000054.1). This high percentage of differentially transcribed, but still poorly annotated genes, is a hindrance to the determination of stage-specific and arthropod-specific virulence determinants of *Rickettsia*. In this study, all of the hypothetical genes expressed by *R*. *felis* in fleas had no other features which might point to a function such as the presence of conserved domains or Blast homology. These genes are best described as genes of unknown function, and experimental evidence is needed for a better understanding of their function in virulence and transmission.

Virulence associated with the expression of LPS biosynthesis machinery has been widely studied in the field of vector-borne diseases. While LPS is recognized by the host immune system, the expression of LPS machinery by bacteria can still confer benefits. Known protective functions of LPS expression include protection from host immune response components such as complement, and the evasion of TLR4 immune recognition through the expression of modified forms of LPS (reviewed in [40]). Modified forms of LPS, specifically hexa- and triacetylated LPS, are expressed by *Yersinia pestis* under conditions most similar to the flea vector [41, 42]. Additionally, virulence genes encoding LPS biosynthesis proteins were necessary for intracellular growth and virulence of *Francisella* in mice [43]. Proteins involved in LPS and membrane remodeling, such as lytic murein transglycosylases encoded by *mltE* [44], have been shown to be upregulated during host infection by *Neisseria meningitidis*, *Erwinia amylovaora* and *Pseudomonas syringae*, aiding in pathogenesis [45]. Additionally, differences in the activity of genes controlling membrane structure in cat fleas correspond with observed morphology of the structural surface components during rickettsial virulence reactivation of tick-borne *Rickettsia* [46]. The upregulation of *lpxB* lipid-A disaccharide synthase, a putative polysaccharide polymerase, and *mltE*, by *R*. *felis* infecting cat fleas suggest a role for LPS synthesis and rearrangement in horizontal transmission by the arthropod host.

Type IV secretion systems and secreted effector proteins have been characterized in intracellular bacteria and aid in growth, infection, and virulence. These secretion systems are present in a diverse set of bacteria, including *Rickettsia*, and function in the transport DNA, conjugation, and the secretion of effector proteins [47, 48]. The *R*. *felis* genome encodes 22 ankyrin repeat-containing ORFs [49], including RF0580 which was upregulated in the current study by both *R*. *felis* str. LSU and *R*. *felis* str. LSU-Lb during infection of fleas. While the function of RF0580 and other ankyrin repeat-containing proteins in *R*. *felis* has not been characterized experimentally, the secretion of ankyrin repeat-containing proteins has been characterized in other Rickettsiales. Secretion of ankyrin repeat-containing protein AnkA through a Type IV secretion system which facilitates intracellular infection in conjunction with tyrosine phosphorylation was identified for *A*. *phagocytophilum* [50]. More recently, the large repertoire of ankyrin repeat proteins (Anks) in *Orientia tsutsugamushi* was determined to be expressed within mammalian host cells and secreted in a heterologous *Escherichia coli* secretion system. Ectopically expressed Anks showed localization to host cell secretion machinery including the endoplasmic reticulum [51]. Together, these studies suggest diverse expression and cellular localization of ankyrin repeat-containing proteins with a wide array of effects including putative virulence functions. Increased expression of ankyrin repeat-containing proteins by *R*. *felis* strains in the flea may aid in increased pathogenesis of the flea host facilitating transmission and virulence; thus requiring further study.

ABC transporters and other membrane synthesis associated proteins are involved in the biogenesis of membrane components and the movement of phospholipids, LPS, and other proteins to the outer membrane of gram-negative bacteria [52]. ATPase binding cassette transporter components such as RF0579 and RF0500 were upregulated in *R*. *felis* str. LSU within flea host. While the function of the four upregulated transporter genes (RF0182, RF0500, RF0579, RF0885) of *R*. *felis* in fleas is not well characterized, ABC transporters have been associated with virulence in flea-borne *Y*. *pestis* [53]. Additionally, ABC transporters have been implicated in the persistence of intracellular bacterium, such as *Salmonella*, by facilitating the movement of effector proteins across the phagolysosome membrane aiding in pathogenesis [54]. In the current study, ATP transporters are highly expressed by *R*. *felis* in fleas. Similar patterns of expression have been previously observed in *Rickettsia conorii* [55] and also in *Ehrlichia chaffeensis* infecting arthropod-derived AAE2 and ISE6 cells [56]. The function of ABC transporters of *R*. *felis* strains in cat fleas is still poorly understood; future studies would be necessary to fully elucidate their involvement in maintenance and transmission of this pathogen.

Another further mechanism of persistence, stress response, and virulence of bacteria is through the expression of toxin-antitoxin systems (TAS). Recent studies have explored the presence of TAS in the genomes of *Rickettsia* spp. and their activity, presenting molecular evidence of apoptotic activity corresponding to bacterial pathogenesis when TAS were overexpressed in *E*. *coli* [57]. Additionally, the presence of toxin-antitoxin genes such as *relB2* and *relE* identified in our RNAseq analyses have been associated with human eschars and vertical transmission of *Rickettsia* in vectors [58]. The RelBE toxin/antitoxin system has been previously characterized in *Mycobacterium* infections, where RelB functions as an antitoxin that neutralizes the toxin RelE. Expression of RelBE has been characterized in *Mycobacterium tuberculosis*, when stress, such as nutrient deprivation, and expression of the RelBE complex together trigger a reduced translational phenotype leading to cell cycle arrest and increased survival of the bacterium [59]. The regulation and function of TAS and their contribution to infection and pathogenesis of the flea vector requires elucidation.

While not previously associated with virulence in other vector-borne systems, genes such as *uvrA and trmE* were expressed in a flea specific manner by both flea- and booklouse-derived *R*. *felis* strains. These genes are involved in the coordination of recombination and DNA repair. UvrA is an exinuclease which aids in repair of DNA. For organisms such as *Mycobacterium*, UvrA, in concert with other repair proteins such as UvrD, recognize and repair DNA damaged by nitric oxide and other host cell response which is a function required for bacteria survival and growth in vertebrate hosts [60]. The gene *trmE* is a GTPase that mainly functions to modify tRNA molecules. A targeted mutagenesis screen identified TrmE of *Francisella tularensis* as a regulator of the expression of genes present on the *Francisella* pathogenicity island involved in sensing changes in nutritional and oxidative stress in eukaryotic cells and regulating genes which induce expression of virulence factors [61]. Genes with defined functions that control the response of *Rickettsia* to environmental stresses experienced during bloodfeeding and transmission may indirectly affect the expression of virulence determinants and aid in successful transmission through known or alternative protein functions.

After qPCR validation of a selected subset of differentially expressed genes in the flea host, seven genes were selected for further transcriptional analysis in the flea infection bioassay. In order to characterize host-dependent transcription of *R*. *felis* during bloodfeeding, fleas were exposed to strains of *R*. *felis* and transcription was assessed and compared to transcription of *R*. *felis* in ISE6 cell culture. Compared to standard *in vitro* culture, expression of selected *R*. *felis* str. LSU genes did not change when incubated in the bovine bloodmeal, however, expression increased in the flea and in the flea feces. These results suggest transcript regulation in *R*. *felis* str. LSU is dependent on interaction with the hematophagous host, specifically the adult flea and fecal stages. The role of fecal transmission has not been well characterized in *R*. *felis* infection, but has been in other flea borne-bacteria [62, 63]. Specifically, *R*. *typhi* fecal transmission by the Oriental rat flea is the primary route of transmission to vertebrates [18]. Viable *R*. *felis* is known to be excreted in the feces of cat fleas, suggesting that fecal transmission could act as an alternative route of transmission to vertebrates and arthropod populations [20]. When combined with the variable rates of *R*. *felis* sustained vertical transmission in cat fleas and co-feeding horizontal transmission, potential non-transovarial, vertical transmission via cat flea larva ingestion of infected adult flea feces may aid in the maintenance of *R*. *felis* in cat flea populations [6]. Regulation of genes which may confer environmental stability to presumably extracellular rickettsiae in cat flea feces could be applied to other insect-borne *Rickettsia*, such as *R*. *typhi* and *R*. *prowazekii*. While functional characterization is required, heightened expression of putative virulence associated genes during flea shedding may point to a greater role of fecal transmission in the overall ecology of *R*. *felis* rickettsiosis.

To support the potential for host-associated transcriptional requirements, the booklouse associated *R*. *felis* str. LSU-Lb expressed *lpxB*, RF0211, RF0580, *virB4_2*, *trmE*, *uvrA*, and *relB2* in a manner similar to the *R*. *felis* str. LSU during the flea infection cycle, increasing transcription in an infection-dependent manner in the hematophagous flea. Expression of the seven target genes suggests a role in transmission in the flea host, as increased transcription is dependent on flea bloodfeeding and is significantly increased as compared to expression in cell culture or in blood alone. The flea imparts pressures far different than those during infection in cell culture or infection in the vertebrate host. Thus, future transcriptional studies must consider vertebrate and arthropod stages, as well as arthropod biology, for a more complete understanding of rickettsial transcription.

Rickettsial strain dynamics may be more complex than is currently understood. Previous assessment of the responses of *Rickettsia* in cell culture without corresponding assessment of transcription in competent vectors may lead to an underestimation of the range of rickettsial activity. To better characterize the dynamic response of *R*. *felis* strains in the hematophagous cat flea and non-hematophagous booklouse, we used RNAseq methodologies to determine and compare the transcription profiles in arthropod hosts. Increased transcription of putative virulence factors including Type IV secretion system factors, LPS synthesis machinery, toxin/antitoxin modules, and proteins in the competent flea vector increases the likelihood for their role in transmission events, providing candidates for targeted experimentation in the cat flea vector. Additional studies are required to determine the complex relationship between *Rickettsia* and arthropod hosts to understand the arthropod-dependent cues responsible for virulence and horizontal transmission.

## Supporting Information

S1 Table *R*. *felis* genes with significant expression in cat fleas as compared to expression in constitutively infected booklice. *Rickettsia* open reading frame (ORF), gene name, gene annotation and Cluster of Orthologous Groups (COG) annotations were retrieved from the URRWXCal2 *R*. *felis* genome (accession CP000053.1, CP000054.1). Log_2_(Fold-change) is reported as the greatest change determined by RNAseq with significant digits determined by the number of reads quantified by Cufflinks. *Expression of *relB2* in booklice was below the limit of detection, but the positive fold change was determined statistically significant by CuffDiff.
(DOCX)
