# Supplementary material for: Differential Rickettsial Transcription in Bloodfeeding and Non-Bloodfeeding Arthropod Hosts
Source: PLoS One. 2016 Sep 23;11(9):e0163769. doi: 10.1371/journal.pone.0163769 (PMC5035074; doi:10.1371/journal.pone.0163769)
Supplement: S1 Table — Rickettsia open reading frame (ORF), gene name, gene annotation and Cluster of Orthologous Groups (COG) annotations were retrieved from the URRWXCal2 R. felis genome (accession CP000053.1, CP000054.1). Log2(Fold-change) is reported as the greatest change determined by RNAseq with significant digits determined by the number of reads quantified by Cufflinks. *Expression of relB2 in booklice was below the limit of detection, but the positive fold change was determined statistically significant by CuffDiff. (DOCX) [file pone.0163769.s001.docx]

**Table S1. *R. felis* genes with significant expression in cat fleas as compared to expression in constitutively infected booklice.**  *Rickettsia* open reading frame (ORF), gene name, gene annotation and Cluster of Orthologous Groups (COG) annotations were retrieved from the URRWXCal2 *R. felis* genome (accession CP000053.1, CP000054.1). Log_2_(Fold-change) is reported as the greatest change determined by RNAseq with significant digits determined by the number of reads quantified by Cufflinks across three biological replicates. *Expression of *relB2* in booklice was below the limit of detection, but the positive fold change was determined statistically significant by CuffDiff.

| ***Rickettsia***  **ORF** | **Log2(Fold- change)** | **Gene** | **Annotation** | **COG** |
| --- | --- | --- | --- | --- |
| **COG: Carbohydrate transport and metabolism** | | | |  |
| RF 0885 | 6.2 | *proP8* | proline/betaine transporter | COG2814G |
| RF 1124 | 3.7 | *emrB* | multidrug resistance protein B | COG2814G |
| RF 0121 | -5.8 | *uhpC* | sugar phosphate permease | COG2271G |
| **COG: Cell cycle control, cell division, chromosome partitioning** | | | | |
| RF 1293 | 4.4 | *Maf* | Maf-like protein | COG0424D |
| **COG: Cell motility, Intracellular trafficking, secretion, and vesicular transport** | | | | |
| RF 0706 | 4.4 |  | P pilus assembly protein FimD, partial | COG3188N |
| **COG: Cell wall/membrane/envelope biogenesis** | | | | |
| RF 0211 | 5.8 |  | LolC/E lipoprotein releasing system, transmembrane protein | COG4591M |
| RF 0519 | 10.8 | *lpxB* | lipid-A-disaccharide synthase | COG0763M |
| RF 0568 | 7.5 |  | hypothetical protein RF_0568 | COG3307M |
| RF 0621 | 3.7 | *mltE1* | soluble lytic murein transglycosylase precursor | COG0741M |
| RF 1000 | 6.2 | *lepA* | GTP-binding protein LepA | COG0481M |
| RF 1036 | 3.1 | *murB* | UDP-N-acetylenolpyruvoylglucosamine reductase | COG0812M |
| RF 1234 | 5.1 | *Pal* | peptidoglycan-associated lipoprotein | COG2885M |
| RF 0006 | -4.0 | *lpxA* | UDP-N-acetylglucosamine acyltransferase | COG1043M |
| RF 0076 | -5.7 | *vacJ* | VacJ lipoprotein precursor | COG2853M |
| RF 0079 | -6.2 | *alr* | alanine racemase | COG0787M |
| RF 0540 | -8.3 | *rffE* | UDP-N-acetylglucosamine 2-epimerase | COG0381M |
| **COG: Coenzyme transport and metabolism** | | | | |
| RF 0036 | 4.2 |  | pyrroloquinoline quinone (coenzyme PQQ) biosynthesis protein C | COG5424H |
| RF 0414 | 4.5 | *ubiG* | 3-demethylubiquinone-9 3-methyltransferase | COG2227H |
| RF 0177 | -2.9 | *coaE* | dephospho-CoA kinase | COG0237H |
| **COG: Defense mechanisms** | | | | |
| RF 1109 | 6.3 | *acrF* | hydrophobe/amphiphile efflux-1 HAE1 family protein | COG0841V |
|  |  |  |  |  |
| **COG: Energy production and conversion** | | | | |
| RF 0029 | 5.9 | *atpE* | ATP synthase F0F1 subunit C | COG0636C |
| RF 0567 | 6.2 | *nuoA* | NADH dehydrogenase subunit A | COG0838C |
| RF 0677 | 6.6 | *gpsA* | NAD(P)H-dependent glycerol-3-phosphate dehydrogenase | COG0240C |
| RF 1009 | 3.1 | *petB* | cytochrome b | COG1290C |
| RF 1010 | 3.1 | *petA* | ubiquinol-cytochrome c reductase, iron-sulfur subunit | COG0723C |
| RF 1092 | 3.2 | *sucA* | 2-oxoglutarate dehydrogenase E1 | COG0567C |
| RF 0136 | -3.2 | *sdhB* | succinate dehydrogenase iron-sulfur subunit | COG0479C |
| RF 0460 | -3.3 | *nuoN2* | monovalent cation/H+ antiporter subunit D | COG0651C |
| RF 0627 | -4.2 | *coxA* | cytochrome c oxidase polypeptide I | COG0843C |
| RF 1161 | -10.2 | *sdhD* | succinate dehydrogenase hydrophobic membrane anchor protein | COG2142C |
| **COG: Inorganic ion transport and metabolism** | | | | |
| RF 0522 | 5.5 |  | frataxin-like protein | COG1965P |
| RF 1325 | 3.6 | *mnhE* | monovalent cation/H+ antiporter subunit E | COG1863P |
| **COG: Intracellular trafficking, secretion, and vesicular transport** | | | | |
| RF 0088 | 3.5 | *virB4_1* | type IV secretion system ATPase VirB4 | COG3451U |
| RF 1250 | 3.6 | *virB4_2* | type IV secretion/conjugal transfer ATPase | COG3451U |
| RF 0089 | -2.7 | *virB6_1* | TrbL/VirB6 plasmid conjugative transfer protein | COG3704U |
| RF 1106 | -2.8 | *ffh* | Signal recognition particle protein | COG0541U |
| **COG: Posttranslational modification, protein turnover, and chaperones** | | | | |
| RF 0692 | -5.8 | *lon* | ATP-dependent protease La | COG0466O |
| RF 1190 | -9.1 | *grxC2* | glutaredoxin-like protein grla | COG0278O |
| **COG: Nucleotide transport and metabolism** | | | | |
| RF 0234 | 3.2 | *Tmk* | thymidylate kinase | COG0125F |
| RF 0709 | 4.1 | *nrdA* | ribonucleotide-diphosphate reductase subunit alpha | COG0209F |
| RF 0770 | 4.3 | *Cmk* | cytidylate kinase | COG0283F |
| RF 0470 | 6.5 | *gppA* | guanosine pentaphosphate phosphohydrolase | COG0248F |
| RF 0339 | -5.2 | *purC* | phosphoribosylaminoimidazole-succinocarboxamide synthase | COG0152F |
| **COG: Amino acid transport and metabolism** | | | | |
| RF 0005 | -2.9 | *gltD* | bifunctional glutamate synthase subunit beta/2-polyprenylphenol hydroxylase | COG0493E |
| RF 0691 | -5.8 | *tdcB* | threonine dehydratase | COG1171E |
| RF 0906 | -3.8 | *panF* | Sodium/pantothenate symporter | COG0591E |
| RF 1143 | -7.7 | *pepA* | leucyl aminopeptidase | COG0260E |
| **COG: Replication, recombination and repair** | | | | |
| RF 0371 | 3.5 | *rickA* | Actin polymerization protein RickA | COG0419L |
| RF 0827 | 1.7e308* | *relB2* | DNA-damage-inducible protein J | COG3077L |
| RF 0999 | 6.2 |  | Transposase | COG3328L |
| RF 1324 | 7.3 | *uvrA* | excinuclease ABC subunit A | COG0178L |
| RF 0530 | -10.7 | *topA* | DNA topoisomerase I | COG0550L |
| RF 0860 | -10.0 | *dnaB* | replicative DNA helicase | COG0305L |
| RF 0926 | -3.3 |  | transposase | COG3328L |
| RF 1295 | -8.1 | *xerC* | site-specific tyrosine recombinase XerC | COG4974L |
| **COG: Transcription** | | | | |
| RF 0305 | -2.5 | *spoT5* | guanosine polyphosphate pyrophosphohydrolase/synthetase-like protein | COG0317K |
| RF 0508 | -6.8 | *spoT2* | guanosine polyphosphate pyrophosphohydrolase/synthetase-like protein | COG0317K |
| RF 0765 | -3.3 | *rho* | transcription termination factor Rho | COG1158K |
| RF 0847 | -2.5 | *icsR* | Iron-sulfur cluster assembly transcription factor IscR | COG1959K |
| **COG: Secondary metabolites biosynthesis, transport and catabolism** | | | | |
| RF 0182 | 3.8 |  | ABC transporter substrate binding protein | COG1463Q |
| **COG: Transcription** | | | | |
| RF 1145 | 6.4 | *rpoC* | DNA-directed RNA polymerase subunit beta\' | COG0086K |
| RF 1146 | 6.4 | *rpoB* | DNA-directed RNA polymerase subunit beta | COG0085K |
| **COG: Translation, ribosomal structure and biogenesis** | | | | |
| RF 0229 | 4.2 | *valS* | valyl-tRNA synthetase | COG0525J |
| RF 0235 | 3.2 | *metG* | methionyl-tRNA synthetase | COG0143J |
| RF 0304 | 6.6 | *rpsT* | 30S ribosomal protein S20 | COG0268J |
| RF 0529 | 2.9 | *gltX* | glutamyl-tRNA synthetase | COG0008J |
| RF 0697 | 2.4 | *yhbH* | sigma(54) modulation protein | COG1544J |
| RF 0722 | 5.7 | *rpsO* | 30S ribosomal protein S15 | COG0184J |
| RF 0871 | 2.6 | *infB* | translation initiation factor IF-2 | COG0532J |
| RF 1278 | 5.6 | *miaB* | (dimethylallyl)adenosine tRNA methylthiotransferase | COG0621J |
| RF 0956 | 5.6 |  | cytotoxic translational repressor of toxin-antitoxin system RelE | COG2026J |
| RF 0721 | -7.3 | *pnp* | polynucleotide phosphorylase | COG1185J |
| RF 1385 | -5.2 | *rpsP* | 30S ribosomal protein S16 | COG0228J |
| RF 1386 | -5.2 | *rpmG* | 50S ribosomal protein L33 | COG0267J |
| **COG: General function prediction only** | | | | |
| RF 0521 | 5.5 |  | hypothetical protein RF 0521 | COG1559R |
| RF 0524 | 5.5 |  | hypothetical protein RF 0524 | COG3975R |
| RF 0579 | 3.5 |  | ABC transporter permease | COG4120R |
| RF 0580 | 3.5 |  | ankyrin repeat-containing protein | COG0666R |
| RF 0698 | 2.4 | *pay* | carbonic anhydrase | COG0663R |
| RF 0711 | 6.4 |  | hypothetical protein RF 0711 | COG1084R |
| RF 1214 | 6.5 | *trmE* | tRNA modification GTPase TrmE | COG0486R |
| RF 0372 | 3.5 |  | tryptophan repressor binding protein | COG0655R |
| RF 0500 | 3.0 |  | ABC transporter ATP-binding protein, partial | COG0488R |
| pRF 11 | -4.9 | *pat2* | patatin-like phospholipase | COG3621R |
| RF 0067 | -4.8 | *sca2* | cell surface antigen Sca2 | COG5271R |
| RF 0233 | -4.8 | *proP4* | proline/betaine transporter |  |
| RF 0474 | -10.2 |  | cell surface antigen-like protein Sca7 |  |
| RF 0626 | -3.1 |  | glutamine amidotransferase | COG2071R |
| RF 0693 | -6.1 | *sca3* | cell surface antigen Sca3 | COG1512R |
| RF 1374 | -3.8 |  | permease | COG0795R |
| RF 1389 | -4.0 |  | oxidoreductase | COG1028R |
| **COG: Function unknown** | | | | |
| RF 0168 | 5.9 |  | hypothetical protein RF 0168 | COG2929S |
| RF 0727 | 2.8 | *Rbn* | ribonuclease BN | COG1295S |
| RF 0178 | -2.9 |  | hypothetical protein RF 0178 | COG3494S |
| RF 0357 | -4.3 |  | hypothetical protein RF 0357 | COG2250S |
| RF 0940 | -7.2 |  | nucleotidyltransferase/HEPN domain-containing protein | COG2250S |
| RF 1068 | -5.2 | *sca8* | cell surface antigen-like protein Sca8 | COG4625S |
|  |  |  |  |  |
| **No COG Assignment** | | | | |
| RF 0169 | 5.9 |  | hypothetical protein RF 0169 |  |
| RF 0170 | 5.9 |  | hypothetical protein RF 0170 |  |
| RF 0183 | 3.8 |  | hypothetical protein RF 0183 |  |
| RF 0184 | 3.8 |  | hypothetical protein RF 0184 |  |
| RF 0386 | 2.8 |  | hypothetical protein RF 0386 |  |
| RF 0520 | 6.5 |  | hypothetical protein RF 0520 |  |
| RF 0523 | 5.5 |  | hypothetical protein RF 0523 |  |
| RF 1215 | 6.8 |  | hypothetical protein RF 1215 |  |
| RF 0077 | -5.7 |  | hypothetical protein RF 0077 |  |
| RF 0108 | -3.0 |  | hypothetical protein RF 0108 |  |
| RF 0233 | -2.5 |  | hypothetical protein RF 0223 |  |
| RF 0356 | -4.3 |  | hypothetical protein RF 0356 |  |
| RF 0507 | -6.8 |  | hypothetical protein RF 0507 |  |
| RF 0688 | -8.4 |  | hypothetical protein RF 0688 |  |
| RF 0925 | -3.3 |  | hypothetical protein RF 0925 |  |
| RF 0937 | -3.8 |  | hypothetical protein RF 0937 |  |
| RF 1160 | -10.2 |  | hypothetical protein RF 1160 |  |
| pRF 02 | -4.1 |  | unknown |  |
